# The performance of levothyroxine tablet is impaired by bariatric surgery

**DOI:** 10.1007/s12020-022-03289-0

**Published:** 2022-12-29

**Authors:** Pierpaolo Trimboli, Nicola Ossola, Alessandro Torre, Francesco Mongelli, Massimo Quarenghi, Chiara Camponovo, Barbara Lucchini, Mario Rotondi, Lorenzo Ruinelli, Fabio Garofalo

**Affiliations:** 1 Servizio di Endocrinologia e Diabetologia, Ente Ospedaliero Cantonale (EOC), Bellinzona, Switzerland; 2 Facoltà di Scienze Biomediche, Università della Svizzera Italiana (USI), Lugano, Switzerland; 3 Servizio di Nutrizione Clinica e Dietetica, Ente Ospedaliero Cantonale (EOC), Bellinzona, Switzerland; 4 Servizio di Chirurgia, Ente Ospedaliero Cantonale (EOC), Bellinzona, Switzerland; 5 Unit of Internal Medicine and Endocrinology, Laboratory for Endocrine Disruptors, Istituti Clinici Scientifici Maugeri IRCCS, Pavia, Italy; 6 Department of Internal Medicine and Therapeutics, University of Pavia, Pavia, Italy; 7 Team Data Science & Research, Area ICT, Ente Ospedaliero Cantonale (EOC), Bellinzona, Switzerland; 8 Clinical Trial Unit, Ente Ospedaliero Cantonale (EOC), Bellinzona, Switzerland

**Keywords:** Hypothyroidism, Bariatric surgery, Levothyroxine, Tablet, Formulations

## Abstract

**Objective:**

The aim was to evaluate if bariatric surgery can affect the LT4 performance. The endpoints were the following: 1) difference between LT4 daily dose before and 1 year after surgery, 2) difference between LT4 dose per weight before and 1 year after surgery, 3) difference among LT4 preparations.

**Methods:**

The study period was between January 2018 and May 2022. Inclusion criteria were a) adults undergone bariatric surgery, b) with proven autoimmune hypothyroidism, c) on LT4 therapy before bariatric surgery, d) using any commercialized LT4 preparation. Excluded were patients a) proven to have or suspected for pre-surgical intestinal malabsorption, b) with other potential interfering factors on LT4 absorption; c) with heart, renal, and/or hepatic failure, d) with recent/current infection/inflammation, e) in pregnancy, f) with incomplete data about LT4 therapy.

**Results:**

According to the selection criteria, 40 patients were included. Both TSH and LT4 daily doses were not significantly different with respect to baseline values. On the contrary, the LT4 dose per weight was significantly increased, especially in RYGB patients. An increased LT4 dose per weight was observed with the reduction of weight.

**Conclusion:**

One year after bariatric surgery 1) the daily dose of LT4 remains unchanged, and 2) despite the significant weight reduction, LT4 dose per weight increases. Most data are referred to LT4 tablet and the performance of LT4 caps should be further investigated.

## Introduction

Hypothyroidism is the insufficient iodothyronines signal inside the target tissues. With the exclusion of rare diseases, three main causes of hypothyroidism can be considered in clinical practice, i.e., 1) (total or not) thyroidectomy, 2) autoimmune thyroiditis (AIT), 3) radioiodine treatment. Despite the cause of hypothyroidism, hypothyroid patient is usually managed by long-life sodium levothyroxine (LT4) therapy [1]. The tablet is the traditional and most largely used LT4 preparation. LT4 tablet is ingested per os in fasting condition, 30 min before having breakfast, and disintegrated and dissolved in the gastric lumen. Importantly, the latter is a critical phase to achieve both optimal activation of active principle and its complete absorption in small intestine [2, 3]. Unfortunately, several factors can interfere with the LT4 tablet pathway [4]. Firstly, gastrointestinal disorders (i.e., Helicobacter pylori gastritis, atrophic gastritis, celiac disease, lactose malabsorption/intolerance) can affect LT4 absorption and hold a major role in clinical practice because of their large worldwide diffusion [5]. Secondly, several drugs (i.e., proton-pump inhibitors, aluminum-containing antacids, calcium carbonate, ferrous sulfate, sucralfate, raloxifene, bile acid sequestrants, phosphate binders), as well as some foods and beverages (mainly coffee and some juices), may reduce LT4 bioavailability. In addition to the above various factors, some papers suggested that bariatric surgery should be considered as further reason of reduced LT4 efficacy to maintain optimal balance of hypothyroidism. The most common bariatric surgeries are Roux-en-Y gastric bypass (RYGB) and sleeve gastrectomy (SG), while other surgical strategies exist, such as biliopancreatic diversion, gastric banding (GB), and jejunoileal bypass (JIB) [6, 7]. Since RYGB and SG are the most diffused, they are the most largely bariatric surgeries investigated as potential interfering factors on LT4. RYGB includes creating small gastric pouch just distal to the gastroesophageal junction and connecting it to the small intestine bypassing stomach, duodenum, and proximal jejunum. With SG, a narrow gastric tube is created along the lesser stomach curve. A systematic review by Gadiraju et al. [8] included ten studies that evaluated the effects of bariatric surgery on LT4 requirements. These studies enrolled patients undergone different surgeries and found that LT4 dose decreased after SG, increased after JIB, and showed heterogeneous findings after other surgical options (including RYGB). A more recent systematic review with meta-analysis [9] was focused on the relationship between thyroid function and bariatric surgery. This review also included studies enrolling patients on LT4 therapy and found that its daily dose decreased after bariatric surgery. However, these findings should be affected by several weaknesses: 1) high heterogeneity among studies was observed, being it unexplained after several sub-analyses; 2) the frequency of reduction of dose or discontinuation of LT4 was mainly associated with SG and RYGB, with unclear reason; 3) the cause of hypothyroidism was unknown in most patients of original studies, which did not allow a conclusion regarding the actual effect of bariatric surgery on primary hypothyroidism (i.e., postoperative or AIT-related); 4) the timing of enrollment of patients after surgery was highly different among studies, with unclear effect of the variations of weight after surgery. Furthermore, no clear information about the correlation between LT4 dose (per day and per weight) and weight and its loss standard measures (e.g., ideal weight, % excess BMI loss, % excess weight loss) was reported. The LT4 absorption has a redundant mechanism and bariatric surgery adds complexity to the system, the size of the literature is small, the population investigated is probably highly selected and observed over a short postoperative follow-up, and most studies focused on RYGB even if more recently SG has been largely diffused. In this context, sparse studies [10, 11] suggested that obese hypothyroid patients on LT4 tablet therapy can have suboptimal TSH after bariatric surgery while experience TSH normalization when switched to LT4 liquid solution (LS) at unchanged daily dose. Then, these studies concluded that this setting of patients could benefit from a liquid formulation. However, the findings from the above studies should be affected by small sample size, different surgeries, timing of enrollment after surgery, and weak indication for LT4. Then, whether LT4 performance is affected by bariatric surgery cannot be proven until now. Also, whether LS or soft-gel caps (SC) of LT4 have actually been considered the optimal preparations in hypothyroid obese patients undergoing bariatric surgery is still unclear.

The present study was conceived to evaluate if bariatric surgery can affect the performance of LT4. Accordingly, a retrospective cohort of hypothyroid obese patients who had undergone bariatric surgery was reviewed. With this aim, the endpoints were the following: 1) difference between LT4 daily dose before and 1 year after surgery, 2) difference between LT4 dose per weight before and 1 year after surgery, 3) difference among LT4 preparations.

## Material and methods

### Institutional management of bariatric patients before and after surgery

The study series included patients undergone bariatric surgery at Ente Ospedaliero Cantonale (EOC), the major public institution in Tessin Canton of Switzerland. EOC bariatric team meets the criteria of Swiss Society for the Study of Morbid Obesity and Metabolic Disorders (SMOB). According to SMOB, a Swiss bariatric center must have a multidisciplinary team with interdisciplinary exchange, including not only the performance of bariatric surgery, but also the corresponding preliminary procedures for the indication of surgery and long-term follow-up of patients (metabolic parameters, malnutrition, psychological care, radiological diagnostics, etc.). EOC bariatric team includes bariatric surgeons, physicians with bariatric expertise (internal medicine/endocrinologist), psychiatrists, psychosomatrists or psychologists with bariatric experience, dietitians with bariatric focus. In addition, dedicated or experienced bariatric specialists such as anesthesiologist, gastroenterologist, cardiologist, pulmonologist, radiologist, pediatrician and adolescent physician, plastic surgeon, physical and exercise therapist, social worker are present. At our institution, according to SMOB, all patients initially selected for bariatric surgery undergo specific clinical work-up to exclude malabsorption disease and nutrients deficit. Particularly, gastroscopy with multiple biopsies is always performed to exclude gastric disease (in primis helicobacter infection and atrophic gastritis), and liver ultrasound is always performed to exclude hepatic disorders. After the operation all patients are treated by pantoprazole at fixed dose of 40 mg per day for 3 months, thromboprophylaxis with low molecular weight heparin subcutaneously (Enoxaparine 40 mg, once per day) for 21 days and multivitamin supplements for at least 12 months. During the postoperative follow-up, the outcome of patients is usually reported according to American Society for Metabolic and Bariatric Surgery (ASMBS) standards [12]. In agreement with this guideline, ideal weight is defined as the weight corresponding to a BMI of 25 kg/m^2^; percent of total weight loss (%TWL) is defined as [(initial weight) – (postoperative weight)]/[(initial weight)] × 100; percent excess BMI loss (%EBMIL) is defined as [ΔBMI/(initial BMI – 25)] × 100; percent excess weight loss (%EWL) is defined as [(initial weight) – (postoperative weight)]/[(initial weight) – (ideal weight)]. Regarding LT4 treatment, at our institution, hypothyroid patients are always asked to ingest it per os in the morning, in fasting condition, 30 min before having breakfast, in the absence of other medications.

### Surgical procedures

The patients enrolled in the present study were submitted to one of three laparoscopic bariatric procedures, such as SG, RYGB, or revisional surgery. SG and RYGB operations were performed according to international standards. In SG, a gastric calibration bougie size 36 or 40 French (Fr) was used. In RYGB, a second anastomosis between the alimentary loop and biliary loop was created at 150 cm from the gastro-digiunal anastomosis. Revisional surgeries included three options: 1) single anastomosis Duodenal-ileal bypass (SADI), a recently reported bariatric surgery resizing of the gastric pouch with distal bypass that falls under the so-called bilio-pancreatic diversions [13]; 2) conversions to RYGB in those patients with persistent reflux disease after SG; 3) gastric pouch resizing with distalization in those cases with either insufficient weight loss either weight regain RYGB [14].

### Study design and patients’ selection

The study period was between January 2018 and May 2022. On June 2022, it started a full retrospective revision of all patients undergone bariatric surgery at our institution with the aim to retrieve data about hypothyroid patients on LT4 therapy before surgery. As inclusion criteria, we considered a) adults undergone bariatric surgery according to SMOB, b) with proven hypothyroidism consequent to AIT, c) on LT4 therapy before bariatric surgery, d) using any commercialized LT4 preparations. Excluded were patients a) proven to have or suspected for pre-surgical intestinal malabsorption, b) with other potential interfering factors on LT4 absorption; c) with heart, renal, and/or hepatic failure, d) with recent/current infection/inflammation, e) in pregnancy, f) with incomplete data about LT4 therapy. A predefined sheet was filled-out to record data about LT4 therapy and weight.

### Levothyroxine formulations

Three different formulations of LT4 were commercially available in Switzerland during the study period, such as tablet (Euthyrox Merck AG and Eltroxin Alfasigma AG), soft-gel (Tirosint IBSA Institut Biochimique SA), and liquid solution (Tirosint solution IBSA Institut Biochimique SA), being the latter one commercialized only since April 2021.

### Laboratory measurement

Thyroid function tests were assessed by Elecsys® e601 Roche Diagnostics. According to the provider’s instructions, TSH has analytical sensitivity of 0.006 mIU/L and reference interval between 0.30 and 4.20 mIU/L, while the reference ranges of free-T4 and free-T3 are 12–22 pmol/L and 3.1–6.8 pmol/L, respectively. Sometimes, both free-T4 and free-T3 are automatically measured only if TSH is skewed.

### Ethics

The present study was included in a larger protocol approved by Tessin Ethical Committee.

### Statistical analysis

All continuous variables were expressed as median and interquartile ranges (IQR). Nonparametric statistical analysis for paired/unpaired data was used. Comparison of paired and unpaired data was performed by Mann–Whitney test. Frequencies were compared by chi-square test. Correlation between two continuous variables was analyzed by linear regression. Statistical significance was set at *p* < 0.05.

## Results

During the study period, a number of 46 hypothyroid patients was treated at our institution with bariatric surgery. Records of these cases were reviewed and after applying the above selection criteria, 40 patients could be included in the study. Baseline features of the study series, and available data, are detailed in Table 1.

**Table 1:** Baseline characteristics of patients included in the study

Feature	Value
Age (years)	48.0 (40.8–57.2)
Female/male (*n*)	36/4
Tablet/SC LT4 preparation (*n*)^a^	31/7
Baseline weight (kg)	112.1 (102.0–126.2)
BMI (kg per m^2^)	42.1 (36.8–47.1)
Ideal weight (kg)	55 (51.4–59.6)
RYGB/SG/revision surgery (*n*)	15/16/9

Continuous variables are expressed as median and IQR

*SC* soft-gel caps, *LS* liquid solution, *RYGB* Roux-en-Y gastric bypass, *SG* sleeve gastrectomy

^a^Data calculated in 40 cases

The results in terms of EWL and EBMIL one year after surgery are illustrated in Table 2. Overall, EWL and EBMIL decreased by 53.9% and 68.2%, respectively, even if revision surgery showed poor outcome. The overall number of cases with %EWL > 50% was 22, being 13/15 after RYGB, 7/16 after SG, and 2/9 after revision surgery. Both loss of weight and BMI were not different between RYGB and SG. Revision surgery showed significantly lower loss of weight and BMI ( < 0.01) with respect to both RYGB and SG.

**Table 2:** Efficacy of bariatric surgeries according to ASMBS standards at 1-year of follow-up

	EWL, %	EBMIL, %
All cases	53.9 (43.9–69.1)	68.2 (57.3–87.8)
RYGB	62.7 (54.0–81.7)	71.2 (68.9–99–9)
SG	50.4 (41.6–64.3)	65.8 (57.2–82.5)
Revision surgery	48.1 (41–51.3)	61.4 (35.4–69.4)

Data are expressed as median and IQR

*ASMBS* American Society for Metabolic and Bariatric Surgery, *EWL* excess weight low, *EBMIL* excess BMI loss

Table 3 illustrates the data of weight, TSH, and LT4 dose one after surgery. Both TSH and LT4 daily dose were not significantly different with respect to baseline values. On the contrary, the LT4 dose per weight was significantly increased in RYGB patients.

**Table 3:** Data of weight, TSH, and LT4 dose collected before and one year after surgery

		All cases	RYGB	SG	Revision surgery
Weight (kg)	Before	112.1 (102.0–126.2)	119.0 (104.0–132.6)	120.3 (96.4–131.6)	98.2 (81.3–108.0)
	After	78.0 (69.5–93.0)	78.0 (69.8–87.1)	85.5 (70.5–100.4)	79.5 (62.2–86)
	*p*	0.000	0.000	0.001	0.103
TSH (mIU/L)	Before	1.5 (0.5–2.6)	1.8 (0.8–4.0)	1.1 (0.1–2.9)	1.1 (0.5–3.0)
	After	2.3 (1.1–4.0)	1.7 (1.4–4.2)	3.0 (1.4–4.1)	2.2 (0.5–3.0)
	*p*	0.290	0.890	0.309	0.505
LT4 daily dose (ug)	Before	100.0 (100.0–150.0)	100.0 (100.0–118.8)	118.5 (100.0–150.0)	100.0 (93.8–131.2)
	After	100.0 (75.0–140.2)	100.0 (81.2–118.8)	116.7 (90.6–150.0)	112.5 (68.8–131.2)
	*p*	0.508	0.734	0.690	0.873
LT4 dose per weight (ug/kg)	Before	1.1 (0.8–1.2)	0.9 (0.7–1.1)	1.1 (1.1–1.3)	1.2 (1.1–1.5)
	After	1.4 (0.9–1.6)	1.4 (1.0–1.5)	1.4 (1.0–2.2)	1.4 (1.1–1.5)
	*p*	0.006	0.022	0.137	0.443

All data are expressed as median and IQR

*RYGB* Roux-en-Y gastric bypass, *SG* sleeve gastrectomy

**Fig. 1:**
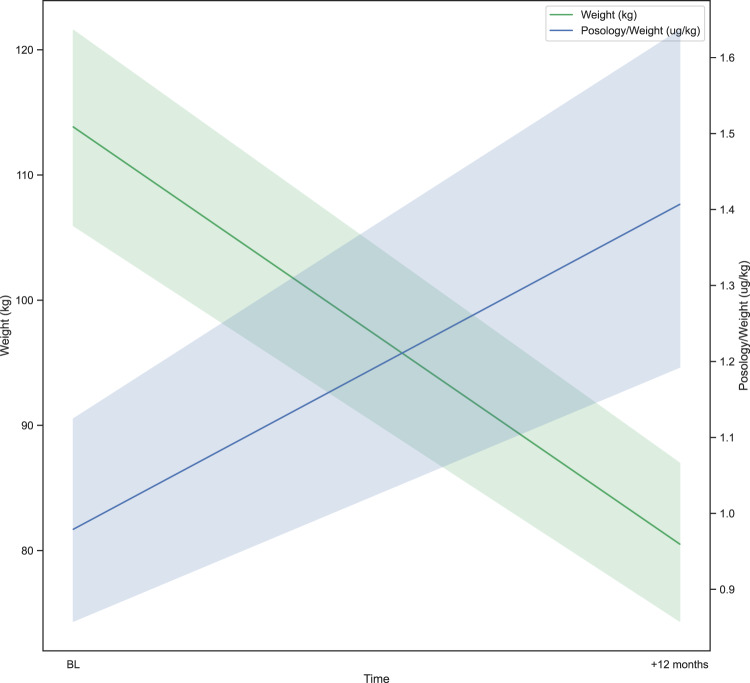
Trend of weight and LT4 dose per weight before and 1 year after bariatric surgery

Several univariate analyses were performed to explain the result of LT4 dose per weight, and it was significantly associated only with total weight loss (*p* = 0.02). As a consequence, no multivariate analysis was attempted. Figure 1 describes the relationship between LT4 dose per weight and weight at baseline and 12-month follow-up. An increased LT4 dose per weight was observed with the reduction of weight.

Following these findings, the potential impact of LT4 preparation was explored. On the one hand, a significant increase of LT4 dose per weight was found in the subgroup of 31 patients using LT4 tablet (*p* < 0.001). On the other hand, no significant variation was observed in the small subgroup of patients on LT4 SC.

## Discussion

This study was aimed at achieving clearer information on the complicated topic of LT4 efficacy in bariatric patients. Currently, no solid data about the performance of LT4 in these patients exist, being obesity associated with several variables with the potential to interfere with the outcome. First, obese patients are intrinsically associated with impaired thyroid function. It is recognized that thyroid gland function suffers from significant increase of body weight. It has not to be overlooked that some studies on this topic enrolled patients with obesity-related hypothyroidism experiencing a consequent resolution of hypothyroidism after bariatric surgery-derived weight loss. The exact mechanism leading to decreased TSH levels following bariatric surgery is not fully understood [9]. Several studies have reported correlation between leptin and TSH levels; the decrease in leptin levels after weight loss, either after diet or after bariatric surgery, represents one main explication of a lower secretion of TSH. In fact, it has been suggested that leptin has a stimulatory effect on thyroid function, which is expressed by increased secretion of TSH [9, 15]. Unfortunately, we could not measure leptin in our patients. Second, the general rule of using a weight-adjusted LT4 dose is less relevant in the obese subjects because it should theoretically apply to lean body mass only [16]. However, lean body mass reduces after bariatric surgery contributing, potentially, to a decreased LT4 dose requirement [17]. Third, bariatric surgery should determine an impaired LT4 absorption with consequent increase of LT4 dose. In this context, it should be cited that, even if RYGB and SG are the most diffused bariatric approaches, alternative options exist with potential different outcome in terms of LT4 absorption [18–21]. Also, it has to be mentioned that SG, performed with different surgical extension, determines achlorhydria that can affect the disintegration process of tablet preparation [22]. Fourth, bariatric patients use several drugs with potential to interfere with the LT4 absorption, especially during the first year after surgery. Indeed, these could represent further confounding factors.

With these premises, this study aimed to answer specific questions, i.e., whether the dose of LT4, daily and weighted-adjusted, changes after bariatric surgery, and whether bariatric patients on different LT4 preparations have different outcomes. Here, 40 patients were selected from our institutional database, their data were extracted, and the efficacy of bariatric surgery was proven according to ASMBS standards [12]. Notably, the enrolled patients were systematically screened for gastrointestinal disorders before surgery. Then, no relevant causes for LT4 malabsorption have to be considered. Therefore, the findings attained merit a discussion.

First, the daily dose of LT4 of our series unchanged after 1 year from bariatric surgery. This result is in line with other reports and corroborates that the topic merits attention from researchers. In clinical practice, both clinicians and surgeons should be aware of this aspect to better tailor the postoperative management of these difficult patients.

Second, beside a significant weight reduction of patients after surgery, an increase of LT4 dose per weight was recorded. Ideally, with the reduction of weight, we should expect a reduction of daily LT4 dose. Since this was not the case, the dose-per-weight ratio increased. This finding could be due to several abovementioned reasons and achieves interest for clinical practice. In fact, regardless of the present specific study setting, we should consider this result when facing patients newly diagnosed with hypothyroidism and having a previous history of bariatric surgery; they will require higher LT4 dose per weight than that expected in general population.

Third, the present data do not allow us to fully answer the question about the different LT4 preparations. Unfortunately, our series included almost only patients on LT4 tablet. This is probably due to the fact that the LT4 SC has been diffused during the last decade and the commercialization of LS in Switzerland occurred on 2021. Anyway, while the LT4 dose per weight was significantly increased in the subgroup of patients on tablet, this was no significant in the small subgroup on SC. The small size of this subgroup does not permit us to address the third study endpoint but can represent an interesting preliminary result requiring further investigations [23]. According to the present findings we can only affirm that the performance of LT4 tablet is impaired by bariatric surgery. Since 2015 American Thyroid Association guidelines suggest to consider non-tablet LT4 preparation in some patients [1], and considering that some papers [10, 11] reported that these preparations can contribute to attain better replacement of hypothyroidism in bariatric patients than the tablet one, the topic merits to be fully explored.

Limitations of this study should be addressed. First, these data are retrospective. Second, the patients included in the study were not managed by one single endocrinology clinic. Third, our series included only autoimmune hypothyroidism. On the other hand, the major strength of the present study is that in almost all patients we had available data at 1 year after surgery, when the weight loss is now over and bariatric patients do not use other medications with potential to interfere the LT4 tablet absorption.

## Conclusions

According to the study aims, the present data allow to conclude that 1 year after bariatric surgery 1) the daily LT4 dose remains unchanged, and 2) despite the significant weight reduction, LT4 dose per weight increases. Most data are referred to LT4 tablet and the performance of LT4 caps should be further investigated.

## Data Availability

All the data used to support the findings of this study are available from the corresponding author upon reasonable request.
